# Parliament and the Profession

**Published:** 1918-04-20

**Authors:** 


### PARLIAMENT AND THE PROFESSION.

Parliament met after the Easter Recess on April 9.

### Vaccination of Troops.

Mr. Macpherson informed Mr. Chancellor that in view of the outbreak of small-pox in London it is not proposed to withdraw the restriction as to leave of men in France who cannot show good vaccination marks.

### The Small-Pox Outbreak.

The President of the Local Government Board stated that of the thirty-four small-pox patients now treated in the hospitals of the Metropolitan Asylums Board eighteen had never been vaccinated and fifteen had been vaccinated or re-vaccinated after exposure to infection.

### Medical Arrangements in Mesopotamia.

Mr. Macpherson gave some details of the arrangements for sick and wounded men in Mesopotamia. These cases are not retained at Ramadi or Hit but are evacuated by motor ambulance convoy or boat to Dhibbam, and thence by ambulance tonga trains to Baghdad, where there is ample hospital accommodation. Medical officers are present with units, field ambulances, and casualty clearing stations, but no nruses for the reasons previously given. Fresh vegetables up to full ration scale have been sent from Baghdad to the troops stationed on the Euphrates, as the quantity available is negligible. While troops are on operations no fruit is issued, but there are ample stocks of dried and tinned fruits in the area.

### Nurses' Kit Allowance.

Sir S. Roberts asked the Financial Secretary to the War Office whether he was aware that, in the case of nursing sisters called for service abroad, the present allowance of £29 for kit is insufficient, and that in many cases these ladies have to spend from £12 to £15 in addition to such allowance to complete their kit; and whether he could take any steps in the matter. Mr. Forster requested Sir S. Roberts to supply him with the figures upon which his question was based, as he could not recognise them.

### Queen Alexandra's Imperial Nursing Service Reserve.

The Financial Secretary to the War Office (Mr. Forster) has stated that the pay and allowances of sisters and nurses of Queen Alexandra's Imperial Nursing Reserve are as follows: Sisters, £50 to £65 per annum; nurses, £40 to £45 per annum. Since November 1, 1916, they have received an addition of £20 a year, if under engagement to serve as long as required. They also receive board and lodging allowance, 25s. a week; uniform allowance, £9 a year; field allowance, 3s. a day; together with accommodation, fuel, and light. For some time, and by error, a money allowance in lieu of lodging, fuel, and light was paid to them, in addition to the provision of lodging, fuel, and light in kind. They were not entitled to it, and it was stopped from February 1, 1916.

